# Apigenin improves cytotoxicity of antiretroviral drugs against HTLV-1 infected cells through the modulation of AhR signaling

**DOI:** 10.1515/nipt-2022-0017

**Published:** 2023-03-25

**Authors:** Dominic Sales, Edward Lin, Victoria Stoffel, Shallyn Dickson, Zafar K. Khan, Joris Beld, Pooja Jain

**Affiliations:** Department of Microbiology and Immunology, Drexel University College of Medicine, Philadelphia, PA, USA

**Keywords:** antiretroviral therapy, apigenin, aryl hydrocarbon receptor, flavonoid, HTLV-1

## Abstract

**Objectives:**

HTLV-1-associated myelopathy/tropical spastic paraparesis (HAM/TSP) is a neuroinflammatory autoimmune disease characterized by high levels of infected immortalized T cells in circulation, which makes it difficult for antiretroviral (ART) drugs to work effectively. In previous studies, we established that Apigenin, a flavonoid, can exert immunomodulatory effects to reduce neuroinflammation. Flavonoids are natural ligands for the aryl hydrocarbon receptor (AhR), which is a ligand activated endogenous receptor involved in the xenobiotic response. Consequently, we tested Apigenin’s synergy in combination with ART against the survival of HTLV-1-infected cells.

**Methods:**

First, we established a direct protein-protein interaction between Apigenin and AhR. We then demonstrated that Apigenin and its derivative VY-3-68 enter activated T cells, drive nuclear shuttling of AhR, and modulate its signaling both at RNA and protein level.

**Results:**

In HTLV-1 producing cells with high AhR expression, Apigenin cooperates with ARTs such as Lopinavir (LPN) and Zidovudine (AZT), to impart cytotoxicity by exhibiting a major shift in IC_50_ that was reversed upon AhR knockdown. Mechanistically, Apigenin treatment led to an overall downregulation of NF-κB and several other pro-cancer genes involved in survival.

**Conclusions:**

This study suggest the potential combinatorial use of Apigenin with current first-line antiretrovirals for the benefit of patients affected by HTLV-1 associated pathologies.

## Introduction

Human T-cell leukemia virus type 1 (HTLV-1) has been identified as the etiologic agent of adult T cell leukemia (ATLL) and the neurological disorder, HTLV-1-associated myelopathy/tropical spastic paraparesis (HAM/TSP) [1]. Despite vigorous cellular and humoral immune responses against HTLV-1 infection, the virus cannot be eliminated due to its ability to integrate its viral genome into its host genome. While most HTLV-1 infections remain asymptomatic, ATLL will occur in 5% of carriers in their lifetime and approximately 2% will develop HAM/TSP [1], [2], [3]. Proviral load is suggested to be an indicator of risk for further development of these disease states, however no distinguishing biomarker has been identified [4]. Current World Health Organization (WHO) guidelines for HTLV-1 infection involve longitudinal observation of infected individuals for the development of ATLL or HAM/TSP but there are no guidelines for treatment. There are little to no efficacious or curative treatments for these disease states; antiretroviral drugs are utilized for their small degree of clinical benefit to patients. However, these therapies can exhibit toxic side-effects with high concentration levels or long-term use. Treatment of ATLL involves the nucleoside reverse transcriptase inhibitor (NRTI) zidovudine (AZT) and interferon-α as the standard first-line of care [5, 6]. HAM/TSP is likewise difficult to treat. Antiretroviral therapy (ART) like AZT have not been demonstrated to alter the course of progression, and management of symptoms mostly involves the use of prednisolone [7]. Viral protease inhibitors are another class of ART that could be applied to HTLV-1 infection. There has been one study evaluating the efficacy of Ritonavir, an HIV protease inhibitor, against HTLV-1-infected cells and primary ATLL cells and it was found that Ritonavir inhibited the activation of NF-κB and led to the induction of apoptosis [8]. Meanwhile, Lopinavir (LPN),another ART that inhibits the HIV-1 viral protease, has the potential to be applied to HTLV-1 infection as well; However, this remains to be tested and explored [9]. Given the current limitations surrounding the treatment of HTLV-1 associated pathologies, it is necessary to explore novel methods of treatment that can address the HTLV-1 infection underlying the development of ATLL and HAM/TSP, as well as to cure these diseases.

Natural and alternative treatments have been an increasingly popular topic of study and have historically been developed into currently used treatments. These natural alternatives provide us with valuable targets of study that may clue into future methods of treatment [10, 11]. One potential avenue of further exploration lies in flavonoids, which are plant compounds derived from a polyphenolic structure, and are ubiquitously found in fruits, vegetables, herbs, and their preparation products including teas and wines. Due to their low molecular weight and hydrophilic nature, these compounds can penetrate the blood brain barrier (BBB) and are observed to have antiviral, anti-carcinogenic, antioxidant, and notably anti-inflammatory effects with relevance to HAM/TSP treatment [12], [13], [14], [15], [16]. Of these compounds, Apigenin has been seen to possess little toxicity even at high doses [17, 18]. Apigenin demonstrates anti-inflammatory, antioxidant, and neuroprotective properties in several cell and tissue systems [19, 20]. As a result, Apigenin has been used to treat many diseases for centuries such as Parkinson’s, neuralgia, shingles, and others (Reviewed in [17]). Likewise, several studies have documented striking anti-carcinogenic effects of Apigenin compared to other flavonoids [21], [22], [23]. Preliminary studies of Apigenin and similar flavonoids have revealed that these effects may be mediated by immune cell response involvement through the modulation of multiple signaling pathways including PI3K/AKT, MAPK/ERK, JAK/STAT, NF-κB and Wnt/β-catenin that play key roles in the development and progression of cancer [22, 24, 25]. Indeed, our own studies of Apigenin have demonstrated that it is able to modulate dendritic cell activity and reduce inflammation in a murine EAE model via the inhibition of the RelB, modulating the inflammatory phenotype [17, 26, 27].

Flavonoids are natural ligands for the aryl hydrocarbon receptor (AhR). The AhR functions as a transcription factor activated by a host of xenobiotic and endogenous ligands including plant flavone and environmental carcinogens, to elicit a multi-pronged anti-toxin response [17, 26, 28], [29], [30]. In its non-activated resting state, the AhR exists in the cytoplasm complexed with multiple chaperone proteins including HSP90, p23, AhR-interacting protein (AIP), and SRC, which keep the AhR in its folded state and prevent degradation [28, 30]. Furthermore, the AhR pathway contributes to the regulation of proliferative and anti-proliferative pathways with immunomodulatory effects, making it a valuable target for the treatment of inflammatory diseases such as MS and in preventing carcinogenesis and cancer treatment [28, 30, 31].

In this study, we tested the hypothesis that treatment with Apigenin activates the AhR-pathway, and this activation leads to an increased sensitivity of HTLV-1-infected immortalized cells to ART. Cellular uptake of Apigenin in PBMCs and HTLV-1 infected cell lines was examined by LC-MS and the interaction between Apigenin and AhR was measured through Nano-ITC. RT-qPCR was used to quantify mRNA expression of targets downstream of AhR-activation under Apigenin treatment and a derivative of Apigenin. We also looked at the protein expression of AhR-regulated targets to support our mRNA findings. The AhR-pathway was screened using a TaqMan array in an HTLV-1 infected cell line after treatment with Apigenin. Cytotoxicity of LPN and AZT in combination with Apigenin, and by first pretreating with Apigenin followed by ART drugs, was determined by the cytotoxicity assay.

## Materials and methods

*Reagents and antibodies*. Apigenin was purchased from Enzo Biochem (New York, NY). VY-3-68 was kindly gifted from Dr. Joseph Salvino from the Wistar Institute (Philadelphia, PA). Antibodies used in this study are AhR (Novus, Littleton, CO), AHRR (Novus), ARNT (Novus), ATF4 (ProteinTech, Rosemont, IL), CYP1A1 (Novus), IDO1 (ProteinTech), Nrf2 (ProteinTech), and β-actin (Cell Signaling, Danvers, MA).

*Cell culture conditions*. HTLV-1 transformed cell line MT-4 [32, 33] was obtained through the NIH AIDS Reagent Program (Catalog No. 120). HTLV-1-negative Jurkat cell line (E6-1, Cat No. 177) was also obtained from the NIH. PBMCs were isolated by Ficoll-Paque density gradient centrifugation on buffy coats from healthy donors (BioIVT, Westbury, NY). All cell lines and primary cells were cultured in RPMI 1640 (Gibco) supplemented with 10% FBS (Biospecialty), 10 mM HEPES (Gibco), and 100 U/mL penicillin-streptomycin (Gibco), and maintained in a humidified incubator with 5% CO_2_ at 37 °C.

*Nano-isothermal titration calorimetry* (*Nano-ITC*)*.* Recombinant human proteins, AhR (LS-G13702-10) and ABCB1 (LS-G142372-20), were purchased from LifeSpan BioSciences. Recombinant proteins were first reconstituted in 50 mM Tris-HCl buffer containing 100 mM glycine at pH 7.2, and then 300 μL of each macromolecule was loaded into the reaction cell of a low volume Nano-Isothermal Titration Calorimeter. The syringe used for the injections contained 50 μL of Apigenin diluted in the same 50 mM Tris-HCl, 100 mM glycine buffer at a concentration of 100 μM and was programmed to inject 2 μL of Apigenin solution every 5 min for both ABCB1 and AhR, respectively.

*Liquid chromatography-mass spectrometry.* Cell pellets from PBMCs treated with Apigenin were collected by centrifugation, washed three times with ice-cold PBS, and 50 μL of ice-cold methanol was used to lyse and extract cellular metabolites. Methanol-cell mixture was incubated for 30 min on ice, centrifuged, and the supernatant collected. The concentration of Apigenin was determined by liquid chromatography mass spectrometry (Waters Acquity UPLC connected to a Waters Synapt G2Si HDMS QTOF) using a calibration curve of the authentic standard as performed in [34].

*RNA isolation and qPCR*. RNA was extracted from cell pellets from various experiments using RNeasy Mini kit (QIAGEN) following manufacturer’s protocol. After RNA quantification, cDNA was prepared using High-Capacity cDNA Reverse Transcription Kit (Applied Biosystems) in a Mastercycler pro S (Eppendorf, Hamburg, Germany). The cDNA was then used for qPCR according to the PowerUP SYBR Green Master Mix protocol (Applied Biosystems) under standard conditions with a QuantStudio 6 Flex Real-Time PCR System (Applied Biosystems). Table 1 list all primers that were used. The 2^−ΔΔCT^ method was used to quantify fold changes in expression of all the genes relative to the control conditions and normalized to the housekeeping gene β-actin.

**Table 1:** Forward and reserves primers utilized in real-time PCR.

Gene	Forward primer	Reverse primer
Ahr	5′TGGTGAAACCCTGTCTCTACTA3′	5′GATTCCCAGGTTCAGGCTATTC3′
Ahrr	5′GCTTCCCACTTCCTTCCTATTC3	5′CCTCCTACCTTGACCTCCTAAA3′
Arnt	5′GGAATGGACTTGGCTCTGTAA3′	5′GTCATCATCTGGGAGGGAAAC3′
Atf4	5′GGAGATAGGAAGCCAGACTACA3′	5′GGCTCATACAGATGCCACTATC3′
Cyp1a1	5′CCAGCTGACTTCATCCCTATTC3′	5′GTAGTGCTCCTTGACCATCTTC3′
Cyp1b1	5′CTGTCTTGGGCTACCACATT3′	5′GGATCAAAGTTCTCCGGGTTAG3′
Ido1	5′CAAAGCAATCCCCACTGTATCC3′	5′ACAAAGTCACGCATCCTCTTAAA3′
Nrf2	5′GTTGCCCACATTCCCAAATC3′	5′CGTAGCCGAAGAAACCTCAT3′

*Protein isolation and western analysis.* PBMCs were either naïve or stimulated with PHA for 24 h. Cells were then spun down to remove PHA and then resuspended in media containing 8 μM Apigenin or 8 μM VY-3-68 and incubated for another 24 h. Cells were pelleted by centrifugation and protein lysates were extracted by resuspending cell pellet with RIPA lysis buffer (Santa Cruz) as per the manufacturer’s protocol. 15 μg of protein was loaded in each lane. Gradient gels were run initially at 80 V for 15 min, after which voltage increased to 120 V for another 1 h. After separation with electrophoresis, the protein was transferred from the gel to a PVDF membrane in a transfer chamber with ice-cold 20% methanol transfer buffer. Transfer was run at 105 V, 4 °C for 1 h. Membranes were blocked at room temperature in 5% non-fat milk powder for 1 h, washed in 0.1% Tween-TBS buffer 3 times for 10 min each, and were then incubated overnight in the primary antibody dilution. Primary antibodies were removed, and membranes washed with 0.1% TBS buffer 3 times for 10 min each, and secondary antibody corresponding to the primary antibody host were added and incubated at 1 h at room temperature. After secondary incubation, membranes were washed with 0.1% Tween-TBS buffer 3 times for 10 min each. Chemiluminescence solution was prepared fresh by combining 1:1 SuperSignal West Pico PLUS (Thermo) and 2 mL was added to membrane and incubated at room temperature for 5 min. Membranes were washed a final time in TBS buffer and imaged on an ImageQuant LAS 4000.

*Flow cytometry.* Cells were harvested from 60–80% confluent cultures by centrifugation for 5 min at 300×*g*. PBS was added to wash cells, centrifuged at 300×*g* for 5 min at 4 °C, and supernatant decanted, three times. Cells were counted and up to 1 × 10^6^ cells/100 mL was aliquoted into FACS tubes containing 500 mL of cold Fixation buffer, vortexed to mix, and incubated for 10 min at room temperature. FACS tubes were then centrifuged at 300×*g* for 5 min at 4 °C, Fixation buffer decanted, and washed once with PBS followed by centrifugation at 300×*g* for 5 min at 4 °C. PBS was decanted and the cell pellet was resuspended in 150 mL of Flow Cytometry Permeabilization/Wash Buffer I. 1 mg of blocking IgG was added per 1 × 10^6^ cells and incubated for 15 min at room temperature. 5 mL of primary antibody was then added to the FACS tube and incubated for 30 min at room temperature in the dark. Cells were washed once with Wash Buffer I, centrifuged at 300×*g* for 5 min at 4 °C, resuspended in 150 mL of Wash Buffer I, 5 mL of secondary antibody added, and incubated for another 30 min in the dark at room temperature. Cells were washed for a final time in Wash Buffer I and resuspended in 300 mL of Flow Cytometry Staining for analysis on FACSCalibur (BD Biosciences).

*Enzyme linked immunosorbent assay* (*ELISA*)*.* Cell supernatants were collected at various time points or drug treatments. ELISA was performed according to the manufacture’s protocol. Briefly, a 96-well plate was first coated overnight at 4 °C with the primary capture antibody. The next day, the plates were washed with assay buffer and then blocked with assay diluent for 1 h with shaking. The plate was then washed 4 times followed by addition of supernatants and standards and incubated at room temperature for 2 h with shaking. The plate was then washed again 4 times, followed by addition of detection antibody and 1 h incubation with shaking. Plate was then washed and Avidin-HRP was then added and incubated for 30 min with shaking. After washing the Avidin-HRP 5 times, TMB substrate solution was then added and incubated in the dark for approximately 15–30 min, followed by addition of stop solution. The plate was then read on a plate reader at 450 and 570 nm.

*TaqMan array.* The mRNA and cDNA used for this array was processed as described before. The TaqMan array (Applied Biosystems) is pre-designed to assay 28 genes associated with the AhR pathway and 4 genes as candidate endogenous controls. The array was carried out according to the manufacturer’s protocol. Briefly, the cDNA first diluted in nuclease-free water such that the final cDNA concentration was 100 ng per well. 20 μL of diluted cDNA was then added to each well and sealed with optical adhesive (Applied Biosystems). Then the array plate was centrifuged for 15 s at 500×*g* and ran similarly as described in prior method.

*MTT viability assay.* The MTT assays were performed according to the manufacturer’s protocol. Briefly, cells were seeded in a 96-well plate at a density of 5 × 10^5^ cells per well in 100 μL of cell culture medium with the tested compounds. 1 mL of PBS was added to the provided MTT vial to make a 12 mM MTT solution. 10 mL of 0.01 M HCl was added to the provided SDS tube on the day of analysis. After 36 h of incubation at 37 °C in 5% CO_2_ incubator, the cells were first centrifuged at 400×*g* for 10 min, then the compound containing media was aspirated and 100 μL of fresh media was added, and 10 μL of the MTT solution was added to each well. Plates were then incubated for 4 h at 37 °C in 5% CO2 incubator. 100 μL of the prepared SDS-HCl solution was then added to each well, and further incubated for 4 h at 37 °C in 5% CO2 incubator. Each well was then mixed with pipette after incubation and measured on a spectrophotometer at 570 nm. For pre-treatment experiments, cells were first plated at (cell concentration) in a 6-well plate. Cell media containing 20 μM Apigenin was used to treat cells overnight. The next day the well plate was centrifuged, Apigenin containing media removed, washed once with PBS, and then fresh media was added. Corresponding treatments were then added after this initial incubation and carried out to their final time points.

*siRNA transfection*. siRNA experiments were conducted according to the manufacture’s protocol for Lipofectamine RNAiMAX. Briefly, cells were plated in a 12-well plate at 2 × 10^5^ density and incubated at 37 °C overnight. The next day, Lipofectamine RNAiMAX was first diluted into a tube containing Opti-MEM medium, then siRNA was diluted into a separate tube with Opti-MEM medium. Diluted siRNA was added to the tube containing diluted RNAiMAX in a 1:1 ratio and incubated at room temperature for 5 min. Afterwards, the 12-well plate was removed from the incubator and the siRNA-lipid complexes were added dropwise to the corresponding well for siAHR or RNAi control. The 12-well plate was then incubated at 37 °C for 36 h and subsequent analysis or experiments followed.

## Results

### Direct interaction of Apigenin with AhR

The model of Apigenin uptake, AhR ligand-receptor binding, AhR nuclear translocation, and transactivation is depicted in Supplemental Figure 1A. As depicted, flavonoids enter cells through passive diffusion and bind to AhR in the cytoplasm. In its resting state, AhR is complexed with multiple chaperone proteins, including heat shock protein 90 (HSP90), p23, AhR-interacting protein (AIP), and proto-oncogene tyrosine-protein kinase Src (SRC). Once engaged with the ligand, AhR dissociates from its chaperone complex and translocates to the nucleus where it dimerizes with ARNT. This dimer is known to then drive the transcription of target genes that contain dioxin-responsive elements (DREs) or xenobiotic response elements (XREs) such as *Ahrr*, *Cyp1a1*, *Nrf2*, and *Atf4*. Different gene sets are activated by distinct AhR ligands that in turn causes a variety of downstream effects.

To test direct binding of Apigenin with AhR, we used nano-isothermal titration calorimetry (Nano-ITC) technique. As a positive control, an ATP binding cassette subfamily B member 1 (ABCB1/P-gp) was utilized, which is a known surface receptor for Apigenin and other flavonoids [35, 36]. Recombinant AhR and ABCB1 proteins were loaded into the reaction cell of a TA Nano-ITC instrument at a concentration of 1 μM and the injection syringe was loaded with 100 μM Apigenin. A comparable binding profile was obtained between Apigenin and its intracellular receptor AhR as seen with the surface ABC transporter protein (Supplemental Figure 1B). The independent modeling on the Nanoanalyze software allowed for the estimation of the dissociation constant (Kd), which for ABCB1 was determined to be 1 nM and for AhR was 4.24 μM.

*Entry and effects of Apigenin on normal PBMCs with respect to AhR signaling genes.* To test the effects of Apigenin exposure on normal blood cells, we first examined cellular uptake of Apigenin through the LC-MS. Methanol extractions of PBMCs treated with 20 mM Apigenin overnight were collected and ran on a Waters Synapt G2Si HDMS QTOF. Apigenin was able to be detected in the treated PBMCs at a concentration of ∼0.2 μM (Supplemental Figure 2). Additionally, treatment of naïve PBMCs with Apigenin overnight resulted in expression of AhR compared to DMSO treatment, suggesting that Apigenin can drive AhR expression in normal PBMCs that generally do not express this protein in the steady state.

**Figure 1:**
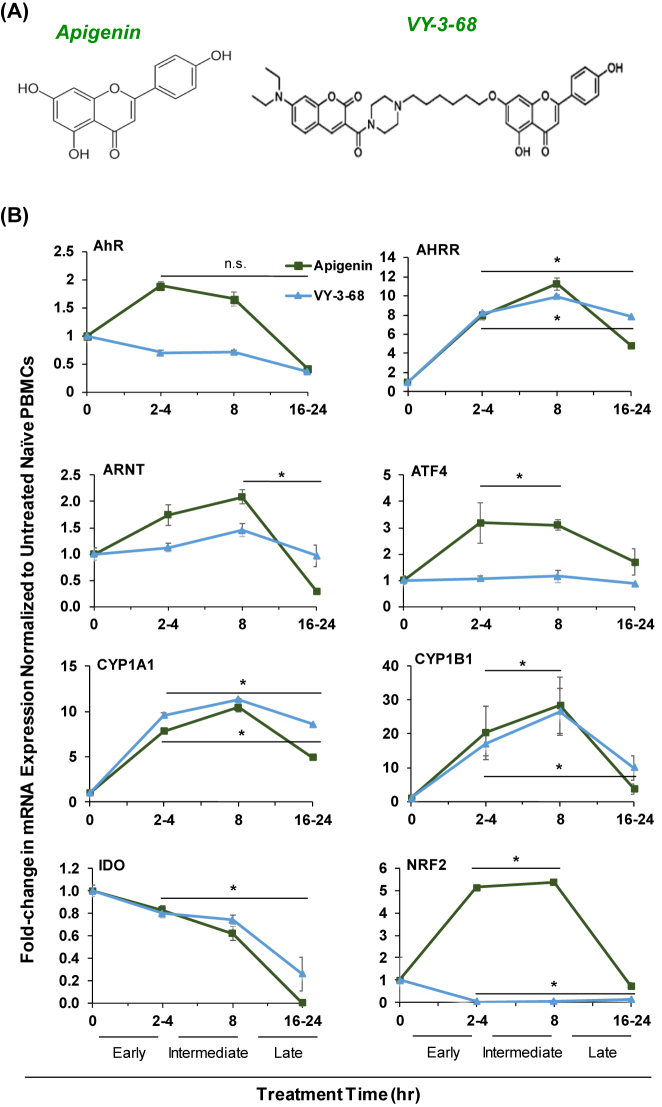
Transient increases in AhR-regulated genes following treatment with Apigenin or its derivative VY-3-68. (A) Chemical structures of Apigenin and VY-3-68 used in these studies. (B) Fold-change in mRNA expression of PBMCs treated with either 8 μM Apigenin or 8 μM VY-3-68 compared to untreated, naïve PBMCs measured by RT-qPCR.

Natural products that display promising benefits for human health can be further chemically modified to achieve increased bioavailability, stability, and solubility, or improve upon their biological effects prior to bringing them into the clinic. One such modified derivative of Apigenin was kindly gifted from our collaborators at Wistar Institute, named VY-3-68 (Figure 1A). Apigenin and VY-3-68 led to mRNA changes in genes of AhR signaling pathway at short, intermediate, and longtime points of 2–4 h, 8 h, and 16–24 h, respectively (Figure 1B). We found that in comparison to untreated, naïve PBMCs, those treated with Apigenin, or VY-3-68 showed significant increase in downstream mRNA transcription. Except for *AhR*, treatment of PBMCs with Apigenin showed a transient increase in mRNA levels of targets downstream of AhR activation. In the case of *AhR*, the levels of mRNA were very slightly upregulated in the short term, but only to an insignificant degree of less than two-fold. Furthermore, the VY-3-68 group showed gradually decreasing levels of mRNA, but once again without any drastic change at any timepoint. *Ahrr*, the primary repressor of the AhR pathway, showed considerable upregulation when stimulated with Apigenin and VY-3-68. A similar trend was observed with the induction of *Cyp1a1*, and with *Cyp1b1*’s upregulation in the Apigenin and VY-3-68 groups. This suggests that Apigenin and VY-3-68 are both able to activate the AhR pathway to a considerable degree for the transcription of *Ahrr* to occur, although to different degrees with varying longevity. Therefore, while the AhR receptor may bind promiscuously to Apigenin and VY-3-68, downstream regulation of gene products by the AhR pathway is differentially activated and repressed depending on the specific ligand. Notably, this effect can be observed in that apart from cytochrome expression, our Apigenin derivative, VY-3-68, elicited lesser degree of effects in *Ahrr*, *Arnt*, and *Ido-1* compared to Apigenin for a more prolonged period. While Apigenin was able to elicit a greater fold change, VY-3-68 demonstrated a more prolonged effective period, as observed in *Ahrr*, *Arnt Cyp1b1*, and *Ido-1* expression. *Nrf2* demonstrates a differential effect between VY-3-68 and Apigenin in the most drastic way; while Apigenin elicited over 5-fold increase in expression, VY-3-68 showed negligible change at all time points. We suggest that variable interaction between AhR and Apigenin/VY-3-68 yield alternative binding affinities of the various AhR-ligand complexes to AhR-binding response elements.

**Figure 2:**
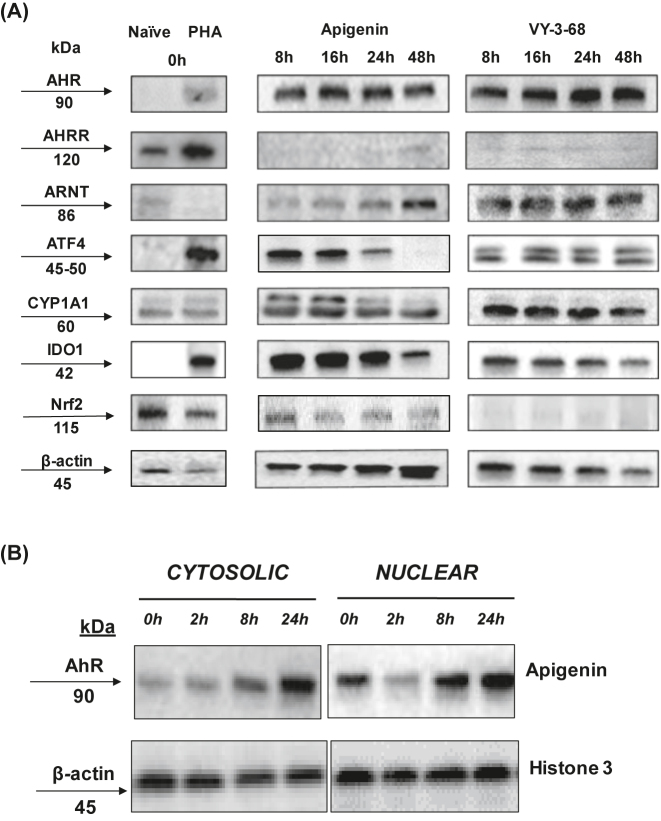
Apigenin treatment modulates the protein expression of AhR-regulated genes. PBMCs were first stimulated with phytohemagglutinin (PHA) for 24 h to activate the T cell population, followed by treatment with either 8 μM Apigenin or 8 μM VY-3-68. Cells were then collected at 8 h, 16 h, 24 h, and 48 h, and subsequently lysed with RIPA lysis buffer. Protein extracts were measured through BCA assay and 15 μg of protein was loaded in each lane.

*AhR activation significantly downregulates the protein expression of CYP1A1 and IDO1 in PBMCs.* Next, we wanted to examine protein expression of known downstream targets of AhR after Apigenin treatment by western blot analysis. Naïve PBMCs and PBMCs activated with PHA were treated with either Apigenin or its derivative VY-3-68 (Figure 2A). AhR protein levels remained constant when treated with Apigenin and its derivative VY-3-68 over the course of 48 hours. No expression is seen for the protein level of AHRR. The AhR nuclear translocator (ARNT) increased protein expression over the course of 48 h when treated with Apigenin, while VY-3-68 showed consistent protein expression. Activating transcription factor 4 (ATF4) demonstrated a decrease in protein expression under Apigenin treatment over 48 hours, while VY-3-68 showed expression but no change. Expression of IDO1 was consistent during the early time points between 8 and 24 h, but there was a decrease in expression by 48 h in the Apigenin and VY-3-68 treated group. For Nrf2, Apigenin treated PBMCs show slight protein expression, while VY-3-68 showed little to no expression. In the case of CYP1A1 the upper band represents a post-transcriptional modification of CYP1A1, which shows decreasing levels of expression over time in all samples despite a robust expression of the lower, pre-modification band.

Since AhR is known to translocate to the nucleus, we also wanted to examine fractionated PBMC lysates to determine cytosolic and nuclear localization of AhR over a time course of 24 h. As seen in Figure 2B, expression of AhR increased in both the nuclear and cytosolic compartments over the course of 24 h. This suggests that AhR treatment not only leads to nuclear translocation but also a total increase of AhR protein within the cell. These results show that treatment of PBMCs with Apigenin results in changes in downstream genes and a translocation of AhR into the nucleus.

*AhR expression and cytokine release in PBMCs.* Additionally, we looked at AhR expression in PBMCs through flow cytometry (Supplemental Figure 3A). In the CD3^+^ populations, we found that 100% of the cells expressed intracellular AhR. For the CD3^−^ populations, we found that 17% of naïve PBMCs expressed AhR whereas PHA-stimulated PBMCs had 24% AhR positive cells. Interestingly, upon treatment with Apigenin there was a reduction in AhR positive cells in the CD3^−^ populations, 13% for naïve PBMCs and 17% for PHA-stimulated PBMCs. The expression of genes involved in inflammatory responses were also measured in PBMCs treated with Apigenin (Supplemental Figure 3B). Gene expression of *Il-12b*, *Ifng*, and *Tnfa* all had a transient peak at 4 hours and then contracted, with *Tnfa* plateauing between 4 and 8 h prior to contracting. The gene *PTGS2* (COX-2) had a sharp decline in expression after Apigenin treatment as early as 4 h. We also examined the level of cytokine secretion in the supernatants of PBMCs treated with either Apigenin or VY-3-68. We saw that IL-2 secretion decreased in both conditions over the same time, with Apigenin treated PBMCs having higher IL-2 expression at 4 h compared to VY-3-68 (104 pg/mL vs 49 pg/mL). IL-10 also increased over this time-period but appeared to plateau after 16 h (Supplemental Figure 3C).

**Figure 3:**
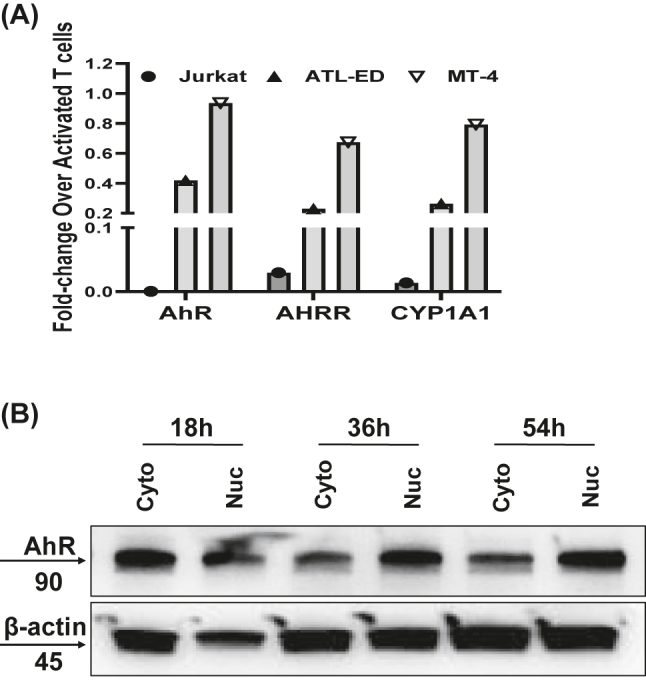
Expression of AhR in HTLV-1 infected cell lines. (A) Fold-change in mRNA expression of AhR and related genes in Jurkat, ATL-ED, and MT-4 cells compared to activated T cells. (B) Determining the cellular localization of AhR in MT-4 cells that were treated with Apigenin over a course of 54 h. Blots show cytosolic and nuclear fractions of MT-4 lysates examining AhR expression in both compartments, with β-actin as the housekeeping protein.

*AhR expression in HTLV-1 infected cell lines and nuclear-cytoplasmic distribution upon Apigenin treatment*. Next, we analyzed AhR expression in HTLV-1 cell lines to examine how AhR levels could be contributing to the functionality of infected cells. To this end, we measured mRNA expression of AhR, AHRR, and CYP1A1 by RT-qPCR in Jurkat cells, ATL-ED cells, and MT-4 cells, and compared this to expression in normal activated T cells (Figure 3A). As previously shown [37, 38], Jurkat had little to no expression of AhR whatsoever, with negligible detection of AHRR and CYP1A1. Compared to activated T cells, ATL-ED had 0.4-fold expression of AhR and 0.2-fold expression of AHRR and CYP1A1, while MT-4 cells had similar expression of AhR (∼1 fold) and CYP1A1 (∼0.8 fold), with a modest reduction in AHRR (∼0.7 fold).

Since it is known that AhR shuttles to the nucleus upon activation to drive transcription, we then wanted to determine the subcellular localization of AhR in HTLV-1 infected cells. MT-4 cells were either left untreated, or treated with either DMSO, Apigenin, or CH223191 (a known antagonist of AhR), and then fractionated into cytosolic and nuclear fractions to determine localization by western blotting. We found that under basal and DMSO conditions, AhR is in both the cytosol and the nucleus (Figure 3B). Upon treatment with Apigenin or CH223191, the nuclear-localized AhR is ablated, and we could only detect AhR expression within the cytosol. This suggests that Apigenin could be functioning as an AhR antagonist like CH223191, however, the cytosolic and nuclear fractions do not exactly match with the total expression. Conversely, Apigenin treatment could result in the degradation of nuclear-localized AhR through the proteasome.

**Figure 4:**
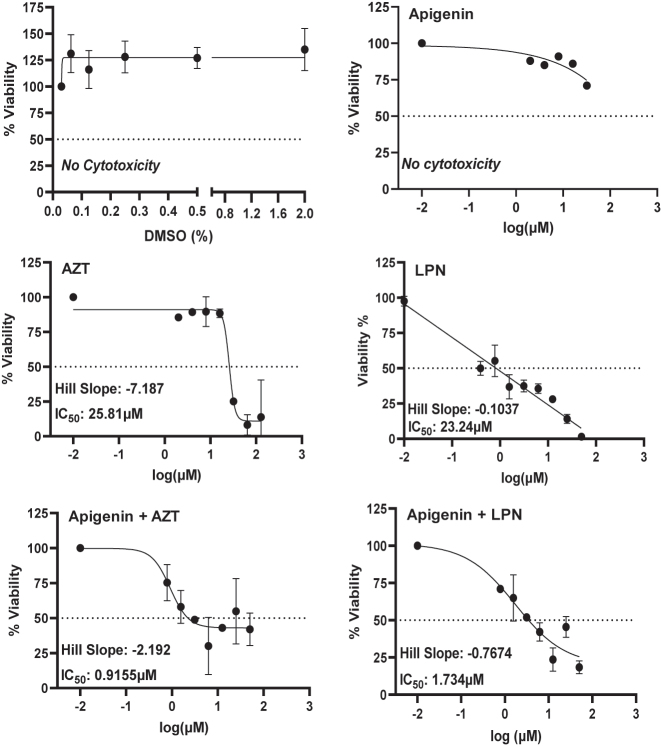
Pre-treatment with Apigenin results in increased cytotoxicity of ART against HTLV-1 infected cell line MT-4. MT-4 cells were seeded at 5 × 10^4^ cells per well in 100 μL of media in a 96-well plate. Compounds were serially diluted in the well ranging from 128 to 2 μM for Apigenin and AZT or LPN alone. For AZT or LPN with Apigenin, media containing 20 μM Apigenin was used to perform serial dilutions. For pre-treatment, MT-4 cells were first treated overnight with 20 μM Apigenin, the next morning the media was removed, replaced with fresh media, then plated followed by serial dilution of AZT or LPN.

*Pretreatment with Apigenin improves cytotoxicity of ART drugs against MT-4.* There have been numerous studies looking at how flavonoids and natural products can be utilized to improve upon currently existing therapies for patients [39], [40], [41], [42], [43]. This improvement can either be accomplished through increased bioavailability of the drug or delayed metabolism, such that there is a prolonged exposure to the drug. We sought to determine how Apigenin could be used to increase the efficacy of existing ART drugs against HTLV-1 infected cells. MTT assays were performed on MT-4 cells to determine IC_50_ values of AZT and LPN (Figure 4). DMSO and Apigenin alone did not result in any observed cytotoxicity at the concentrations tested (0–2% for DMSO, and 2–128 μM for Apigenin). Next, we tested AZT and LPN alone against MT-4 cells and found that AZT had an IC_50_ of 25.81 μM whereas LPN had an IC_50_ of 23.24 μM. Then we repeated this experiment and included an overnight incubation with 20 μM Apigenin prior to conducting the MTT assay. We found that pretreating MT-4 cells with Apigenin resulted in a significant decrease in IC_50_ values for both drugs, 0.916 and 1.73 μM for AZT and LPN, respectively. These results demonstrate that Apigenin can be used to increase the cytotoxicity of existing ART drugs *in vitro*.

**Figure 5:**
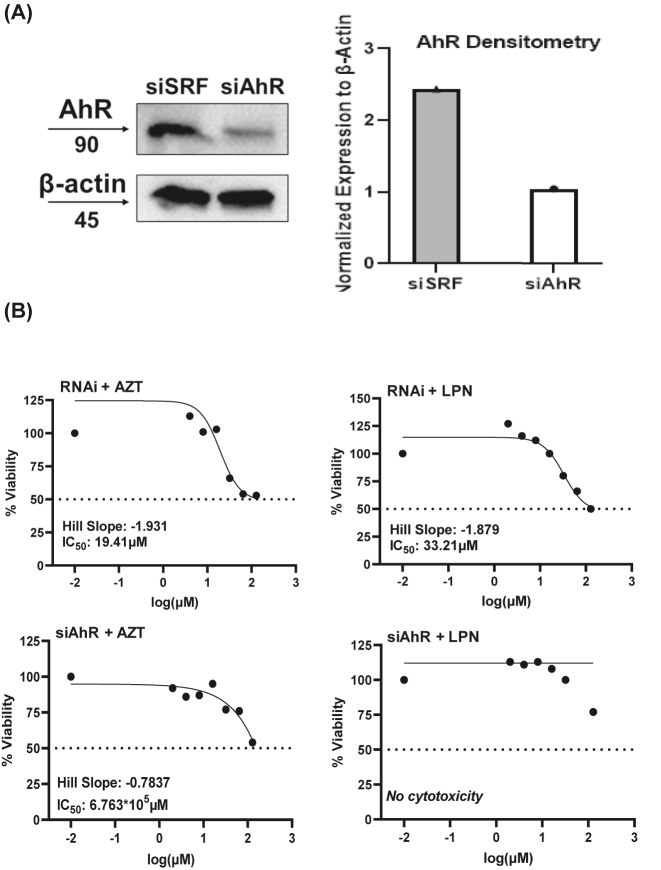
siRNA knockdown of AhR decreases cytotoxicity of ART against MT-4 cells. (A) Knockdown of siAhR in MT-4 cells. MT-4 cells were treated with siRNA for 36 hours and blotted for AhR. Densitometry of the western blot is on the right. (B) MT-4 cells were treated with siRNA for 36 hours and then seeded at 5 × 10^4^ cells per well in 100 μL of media in a 96-well plate. Compounds were serially diluted in the well ranging from 128 to 2 μM for both LPN and AZT.

*Knockdown of AhR leads to increased ART cytotoxicity.* To demonstrate that our observations of increased ART cytotoxicity is AhR-dependent, we used siRNA knockdown of AhR (siAhR) and measured cell viability through MTT with ART treatment. MT-4 cells were first plated in 12-well plates, incubated overnight, siRNA complexes added the next morning, and then allowed 36 h for siRNA knockdown. First, we checked to see the efficiency of the knockdown, and found that siAhR resulted in a 57% decrease in expression compared to the siSRF control (Figure 5A). Next, the cells treated with siAhR, and RNAi reagent as a control, were then collected, plated into a 96-well plate, and then ART was serially diluted as performed previously. MT-4 cells that were treated with RNAi control had a similar IC_50_ value compared to ART treatment alone for both AZT (25.81 vs. 19.41 μM) and LPN (23.24 vs. 33.21 μM). Interestingly, siAhR treatment of MT-4 resulted in a much higher IC_50_ value for AZT and was unable to calculate an IC_50_ value for LPN with the same drug range tested for RNAi (Figure 5B).

**Figure 6:**
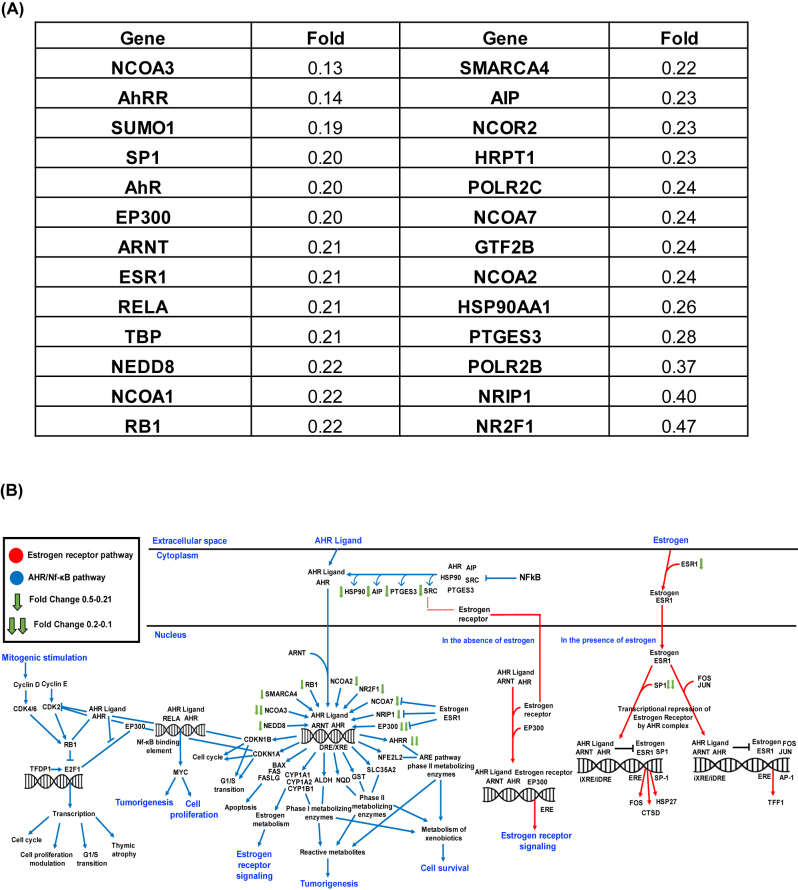
Effect of Apigenin treatment on the AhR pathway(s) in HTLV-1. (A) Table of the most highly downregulated genes in MT-4 cells treated with Apigenin compared to DMSO in a TaqMan AhR array. (B) Ingenuity analysis pathway diagram depicting the network of proteins and genes that are involved in AhR-signaling, are a product of AhR-signaling, and those that interact with AhR.

*AhR pathway genes are downregulated in HTLV-1 infected cells upon Apigenin treatment.* To further examine the effect of Apigenin on the AhR pathway in the context of HTLV-1, a TaqMan array designed for 28 AhR-related genes and targets was used to quantify RNA extracted from MT-4 cells treated overnight with Apigenin or DMSO as a vehicle control. Surprisingly, all the significant differentially expressed genes were downregulated under Apigenin treatment (Figure 6A). Some of the genes that were most highly downregulated include *Ncoa3* (0.13-fold), *Ahrr* (0.14-fold), *Sumo1* (0.19-fold), *Sp1* (0.2-fold), and *Ahr* itself (0.20-fold). The genes included in this dataset were uploaded into QIAGEN’s Ingenuity Pathway Analysis (IPA) software. Pathway analysis revealed that AhR interacts with various signaling pathways involved in cell cycle progression, cellular proliferation, apoptosis, and tumorigenesis as depicted in Figure 6B.

## Discussion

HTLV-1 remains a neglected tropical disease without a vaccine and with the potential to develop into HAM/TSP or ATLL. HTLV-1 is endemic to multiple regions of the world including Japan, sub-Saharan Africa, South America, and the Caribbean, and is spread via sexual contact, contaminated blood, and from mother to child [44]. With a lack of global awareness and testing protocols, HTLV-1 is likely spreading unknowingly and could result in an increase of HTLV-1 associated pathologies. Thus, it is imperative that treatment options be improved upon, and public health strategies be employed to provide better outcomes for patients with HAM/TSP or ATLL and to inform populations of potential HTLV-1 spread in their communities.

Flavonoids and natural products have been used for thousands of years to aid in the treatment of various inflammatory conditions, such as infection and cancer. Apigenin is one of the flavonoids that has been widely used and studied, due in part to its low cytotoxicity and long history of use in treating diseases. Flavonoids have been shown to interact with the AhR and their specific chemical structures can lead to differential cellular responses [45]. In this study, we provided confirmation of interaction of Apigenin with AhR as a direct ligand. Thereafter, we investigated the effect of Apigenin-AhR binding on the mRNA and protein expression profiles of PBMCs. Our studies demonstrate that within PBMCs, a similar pattern of AhR activation can be observed by stimulation of the AhR by Apigenin and its derivative compound, VY-3-68, although differences in downstream mRNA and protein expression profiles between the two compounds suggest that the two compounds may interact differently with the AhR to potentiate their respective effects. Several papers have posited that AhR ligands are able to selectively activate the AhR pathway to differing degrees and may even be capable of causing differential activation of downstream components of the AhR activated response [45, 46], lending credibility to this potential finding. As observed in the mRNA expression of *ahrr, cyp1a1, cyp1b1,* and *ido*, VY-3-68 can stimulate expression of these downstream effectors to nearly the same degree as Apigenin, or to an even greater degree as seen in *cyp1a1* expression. Meanwhile in *atf4* and *nrf2*, the same trend is not observed. Jin et al. demonstrated that the number of -OH groups in any given AhR ligand was able to predict the degree of AhR interaction [45]. In Figure 1A, we can see that Apigenin displays more exposed –OH groups, potentially causing its greater effects on mRNA expression, especially on *atf4* and *nrf2*. The roles of these genes are also of interest. ATF4 was identified to be capable of Tax transactivation of HTLV-1; thus the observable uptick in mRNA expression after Apigenin stimulation may be a clue to the potential Apigenin has in augmenting ARTs [47]. Despite a blunted response, our results show that VY-3-68 demonstrates a more prolonged effect on AhR activation, maintaining a greater degree of mRNA transcription at longer timepoints. This could potentially be due to the extra side chains added onto Apigenin in the formulation of VY-3-68, which could lead differences in interaction with AhR and differences in metabolism of this compound.

The role of AhR in regulating immune cell differentiation and the release of cytokines has also been documented. It was found that exposure to different AhR ligands can skew the resulting T-cell populations towards regulatory T (T_reg_) cells or towards interleukin-17 (IL-17) producing CD4^+^ T helper cells (T_H_17) [48]. AhR has also been found to be critical in controlling monocyte differentiation between dendritic cells and macrophages, further supporting AhR’s role in immune regulation [49]. It has also been reported that AhR activation can have antagonistic effects on TNF-α signaling [50]. In agreement with this, our results show that treatment of PBMCs with Apigenin decreased TNF-α expression at the mRNA level.

There has also been increased interest in understanding how AhR can impact anti-viral immunity [51]. Similar to what has been described in AhR’s regulation of differentiation in T cell populations, the role of AhR during viral infection can have both positive and negative consequences in host immune responses mounted against the virus [52]. It has been previously shown that AhR is highly-upregulated in some patients diagnosed with ATLL [53].

Drug combinations have been successfully utilized in the clinic to improve efficacy of treatment when used by themselves. A computational study that looked at the pharmacokinetic properties of ART compounds in combination with dietary flavonoids suggested that Apigenin could be utilized to increase the efficacy of LPN, however this was never confirmed *in vitro*. Our data from MTT experiments is in support of their computational findings. Compared to ART treatment alone, pretreatment with Apigenin resulted in a significant decrease in IC_50_ values from 25.81 to 0.916 μM and 23.24–1.73 μM for AZT and LPN, respectively. This suggests to us that Apigenin either sensitizes these cells to ART or Apigenin is interacting with AhR in such a manner that leads to increased cytotoxicity in MT-4 cells. Further support for this notion comes from our siRNA experiments that showed knockdown of AhR resulted in decreased cytotoxicity of MT-4 cells treated with ART.

In respect to the AhR TaqMan array, the genes involved in this assay include those that AhR is known to interact with like regulatory and signaling pathways, as well as pathways mediated by the estrogen receptor (ESR) and nuclear factor-κB (NF-κB). Some of the most highly downregulated genes from the assay such as *Ncoa3*, *Sumo1*, *Sp1*, *Ahr*, and *Ep300* are highly expressed in these pathways. It has been shown that persistent activation of the ESR and NF-κB signaling pathways promote uncontrollable cellular proliferation [54, 55]. It is therefore not surprising that both ESR and NF-κB signaling pathways are upregulated during HTLV-1 infection [56, 57]. Our data show that Apigenin downregulates these genes sensitizing MT-4 cells and contributing in the enhancement of ART efficacy.

Subsequently, the genes detected in the TaqMan array were input into QIAGEN’s IPA software, which revealed that AhR communicates with various tumor suppressors and proto-oncogenes. These genes along with their signaling pathways are involved in cell cycle progression, cellular proliferation, apoptosis, and tumorigenesis. AhR mediates its effects through interactions with proteins such as NF-κB, ESR, and retinoblastoma protein (RB). Direct association between AhR and RelA promotes the transactivation of c-Myc protein which is involved in cellular proliferation and tumorigenesis. Conversely, the AhR complex inhibits ESR signaling in the presence of estrogen that would otherwise normally transcribe genes such as FOS, CTSD, HSP27, and TFF1, which are over-expressed and/or mutated in various cancers. Likewise, the interaction between AhR and RB represses E2F-responsive genes that are involved in cell cycle progression. Since Apigenin was able to suppress genes involved in theses pathways, the downstream effects may have resulted in the downregulation of cytotoxic effects arising from the ARTs. This is most likely what led to the improved IC_50_ values when Apigenin was used in combination with ARTs.

Together, our results demonstrate that Apigenin can be used to modulate AhR activity and increase efficacy of ART drugs against HTLV-1 infected cell lines. Apigenin was shown to be efficiently taken up by cells *in vitro*, and treatment with Apigenin resulted the shuttling of cytosolic AhR into the nucleus accompanied with transient increases in expression of AhR-regulated genes, like *Cyp1a1*, *Cyp1b1*, and *Ahrr*. Flow cytometry revealed that all CD3^+^ populations in PBMCs expressed intracellular AhR. MT-4 cells showed similar expression of *AhR* compared to activated T cells, as well as the AhR-activation read out gene *Cyp1a1*, suggesting that HTLV-1 infected cells have constitutively active AhR. Pretreatment of MT-4 cells with Apigenin resulted in a marked increase in cytotoxicity for both LPN and AZT, and this effect was blunted upon knockdown of AhR with siRNA. Apigenin treatment of MT-4 cells showed a downregulation of many genes involved in ESR and NF-κB, both of which are implicated in proliferation of HTLV-1 infected cells, suggesting that Apigenin can “prime” these cells for elimination by ART.

## Supplementary Material

Supplementary Material Details

Supplementary Material Details

Supplementary Material Details

Supplementary Material Details
