# Supplementary material for: Apigenin improves cytotoxicity of antiretroviral drugs against HTLV-1 infected cells through the modulation of AhR signaling
Source: NeuroImmune Pharm Ther. 2023 Feb 17;2(1):49–62. doi: 10.1515/nipt-2022-0017 (PMC10070013; doi:10.1515/nipt-2022-0017)
Supplement: Supplementary file 1 — Supplementary Material Details [file j_nipt-2022-0017_suppl_001.docx]

Supplementary Figure Legends

Supplemental Figure 1. **A)** Apigenin enters the cell and serves as a ligand for AhR. Model figure depicting flavonoid entry into the cell. Flavonoids, like Apigenin, can passively diffuse through the membrane. AhR resides in the cytoplasm in an inactive state by complexing with its chaperone proteins AhR interacting protein (AIP), heat shock protein 90 (HSP90), p23, and proto-oncogene tyrosine-protein kinase Src (SRC). AhR is activated upon ligand binding, which results in a conformational change, dissociation of its chaperone proteins, and exposure of a nuclear localization signal, which allows for nuclear shuttling of AhR. In the nucleus AhR will dimerize with AhR nuclear translocator (ARNT, also known as HIF-1β) and bind to promoter regions of genes containing an AhR responsive element (ARE) to drive transcription. B) Exchanges in heat over time between Apigenin and either AhR or ABCB1 measured by Nano-isothermal titration calorimetry (Nano-ITC). 100 μM solution of Apigenin was loaded into the syringe and 1 μM solution of either recombinant-AhR or recombinant-ABCB1 was loaded into the experimental cell. 2 μL from the syringe loaded with Apigenin was injected into the experimental cell every 5 min.

Supplemental Figure 2. A) Intracellular concentration of Apigenin in PBMCs treated overnight with 20 μM Apigenin measured by LCMS. B) Western blot for AhR of naïve PBMCs treated with 20 μM Apigenin overnight, with β-actin as loading control.

Supplemental Figure 3. A) Intracellular staining of AhR measured by flow cytometry of naïve and PHA activated PBMCs. B) mRNA expression of inflammatory genes in PBMCs treated with Apigenin over 24 h measured by qPCR. C) Cytokine secretion in PBMCs treated with Apigenin or VY-3-68 over 48 hours.
