# Supplementary figures and images for: Apigenin improves cytotoxicity of antiretroviral drugs against HTLV-1 infected cells through the modulation of AhR signaling
Source: NeuroImmune Pharm Ther. 2023 Feb 17;2(1):49–62. doi: 10.1515/nipt-2022-0017 (PMC10070013; doi:10.1515/nipt-2022-0017)

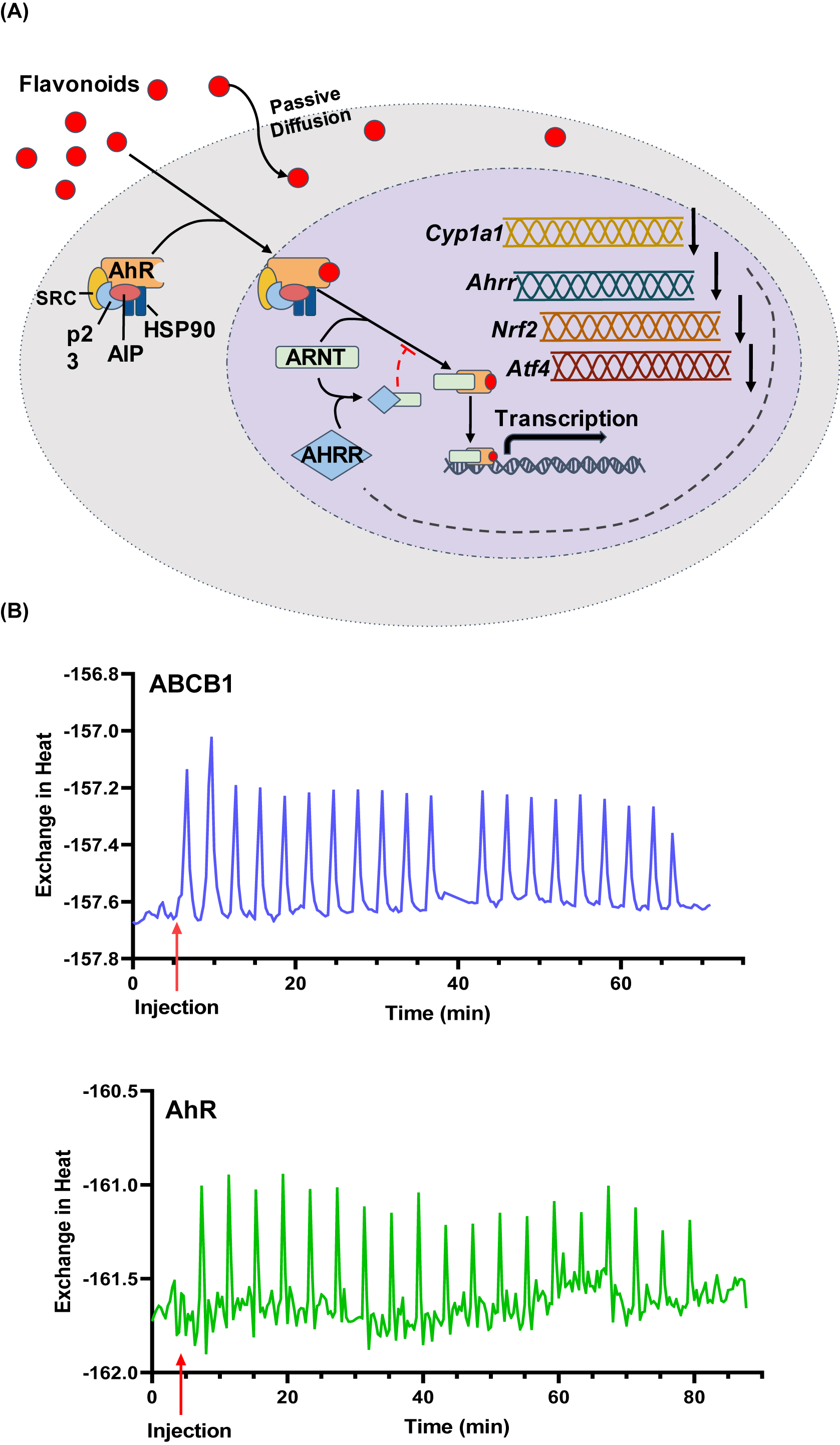

Supplement: Supplementary file 2 — Supplementary Material Details [file j_nipt-2022-0017_suppl_002.jpg]

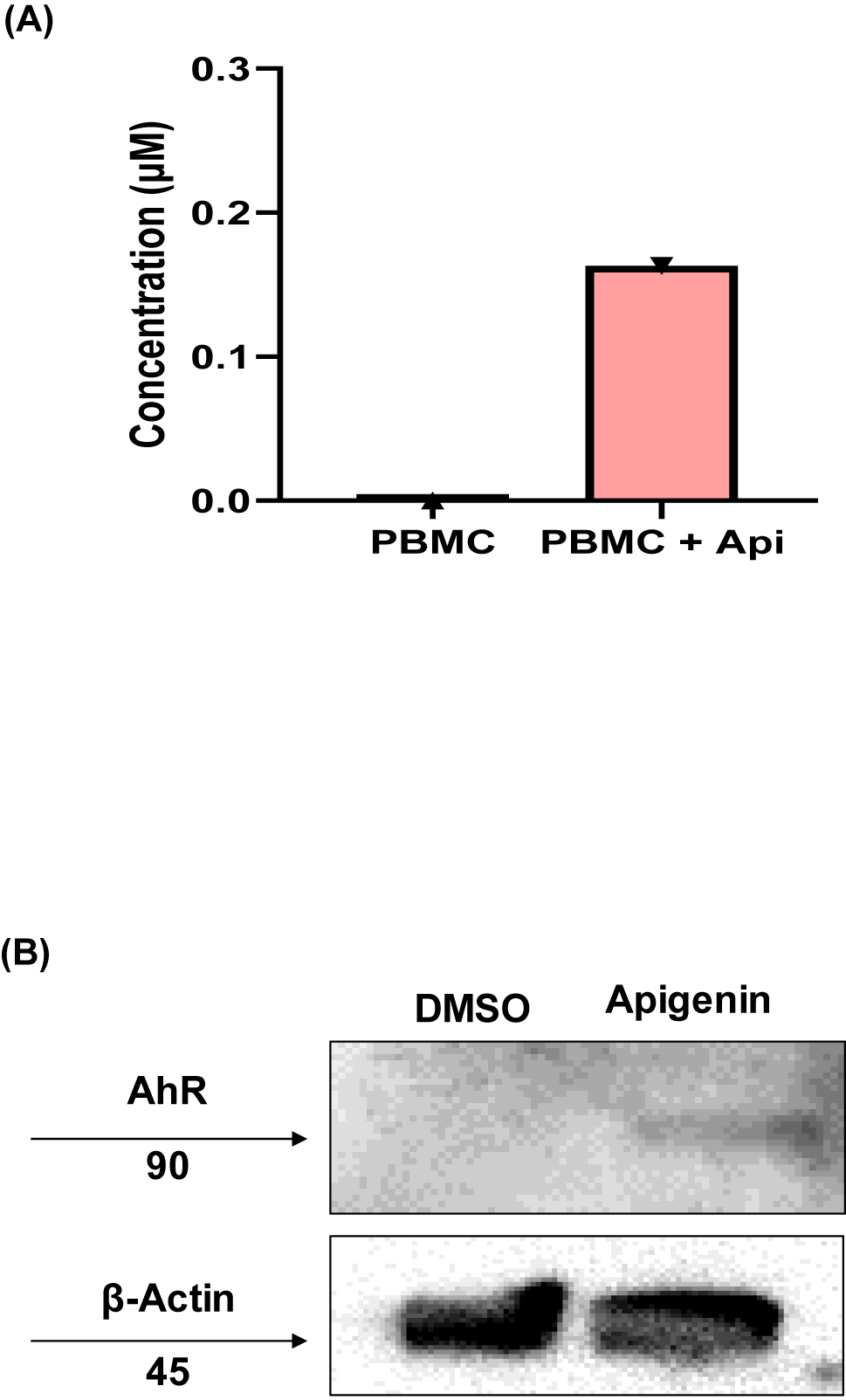

Supplement: Supplementary file 3 — Supplementary Material Details [file j_nipt-2022-0017_suppl_003.jpg]

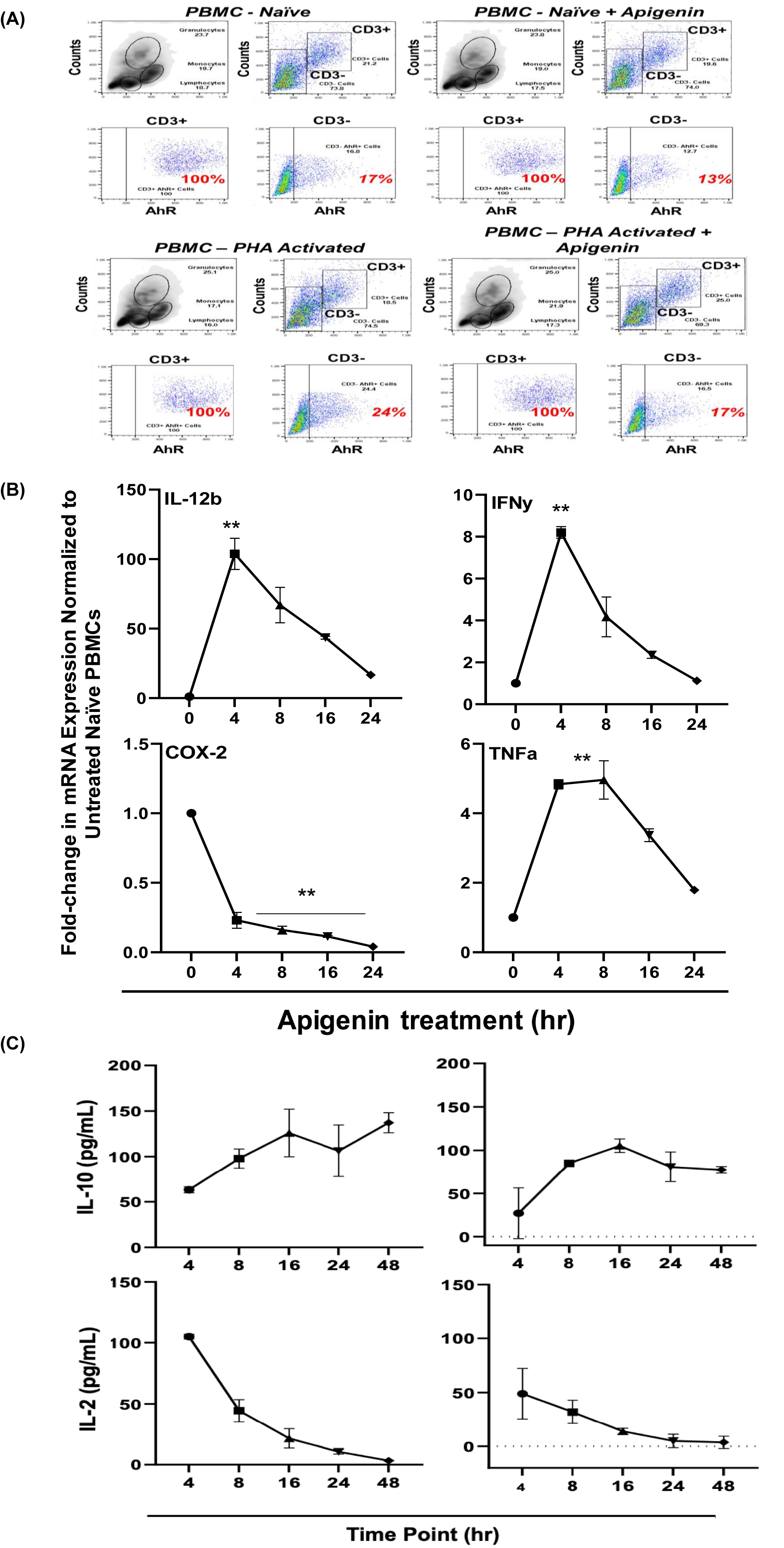

Supplement: Supplementary file 4 — Supplementary Material Details [file j_nipt-2022-0017_suppl_004.jpg]
